# Powdered tart cherry supplementation effectively reduces markers of catabolism and perceptions of muscle soreness following an acute bout of intense endurance exercise

**DOI:** 10.1186/1550-2783-11-S1-P33

**Published:** 2014-12-01

**Authors:** E Galvan, K Levers, R Dalton, C Goodenough, A O’Connor, S Simbo, N Barringer, J Carter, C Seesselberg, A Coletta, YP Jung, M Koozehchian, B Sanchez, S Springer, M Cho, S Mertens-Talcott, C Rasmussen, M Greenwood, R Kreider

**Affiliations:** 1 Texas A&M University, College Station, Texas, USA

## Background

Consumption of tart cherry juice has been reported to effectively reduce inflammation, muscle damage, and muscle soreness following bouts of exercise. The purpose of this study was to determine if consumption of a powdered form of tart cherries derived from tart cherry skins prior to and following intense endurance exercise promotes similar positive results as seen with tart cherry juice consumption.

## Methods

27 endurance trained or triathlete (21.8±3.9 yr, 15.0±6.0% body fat, 67.4±11.8 kg) men (n=18) and women (n=9) volunteered to participate in this study and were matched based on average reported race pace, age, body weight, and fat free mass. Subjects were randomly assigned to ingest, in a double blind manner, capsules containing a placebo (P, n=16) or powdered tart cherries (CherryPURE® Freeze Dried Tart Cherry Powder [TC, n=11]). Participants ingested the supplements one time daily (480 mg/d) for 10-d including day of exercise up to 48-hr post-exercise. Rate perceptions to a standardized application of pressure via an algometer on their dominant thigh at 3 designated locations using a 10-point visual analogue scale were implemented to assess muscle soreness/tenderness over the course of the testing protocol. A half-marathon run (13.1 mi/21.1 km) was completed under 2-hr (111.98±11.9 min) as the intense endurance exercise protocol. Fasting blood samples and VAS ratings of muscle soreness were taken pre-run, 60-minutes post-run as well as after 24 and 48 hours of recovery and analyzed by MANOVA with repeated measures. Consent to publish the results was obtained from all participants.

## Results

Pain ratings from all 3 quadriceps locations (p<0.001); AST, ALT, CK, BUN/Cr ratio, UA (p<0.001); cortisol, testosterone, and CORT/TEST ratio (p<0.001) all demonstrated significant changes in both groups over time. The overall MANOVA analysis revealed a trend toward a significant group x time effect for all pain ratings (p=0.093) and cortisol, testosterone, CORT/TEST ratio (p=0.071), but no significant interaction MANOVA Wilks’ Lambda effects were seen for AST, ALT, CK, BUN/Cr ratio, UA (p=0.365). MANOVA univariate analysis revealed significant effects for v. medialis [¼] (p=0.003) and v. lateralis [½] (p<0.001) pain perceptions, AST, ALT, UA, cortisol (p<0.001), CK (p=0.001), BUN/Cr ratio (p=0.008), and cort/test ratio (p=0.042) in both groups over time in addition to trends for both groups over time for v. lateralis [¼] pain perception (p=0.12) and testosterone (p=0.08). A significant group x time linear effect was shown for v. medalis [¼] pain perception (p=0.035) with a trend toward a significant quadratic interaction for the v. lateralis [½] pain perception (p=0.053). A significant delta value was also shown based on v. medialis [¼] pain perception differences in group assignment (p=0.029). No significant group x time interaction was evident in pain perception from the v. lateralis [¼] (p=0.92). A significant group x time quadratic effect was shown for BUN/Cr ratio (p=0.048). No significant group x time interaction was evident for ALT (p=0.70), AST (p=0.92), CK (p=0.87), and UA (p=0.50). The MANOVA univariate analysis also revealed a significant group x time effect for cortisol (p=0.05) coupled with a more specific group x time significant cubic (p=0.009) and linear (p=0.016) interactions for cortisol.

## Conclusion

Results of this study indicate that acute supplementation with powdered tart cherries over the 7 days leading up to, during, and 2 days after intense endurance exercise helps to minimize post-training perceptions of pain in the most biomechanically loaded regions of the quadriceps muscle group associated with running impact compared to a placebo. Additionally, powdered tart cherry supplementation is also proven to help attenuate the catabolic response of cortisol following a bout of intense endurance exercise. This attenuation of the catabolic response following the acute bout of endurance exercise is substantiated by a significantly lower BUN/creatinine ratio in the powder tart cherry group versus those supplementing with the placebo. Overall, these findings suggest that supplementation with a powdered tart cherry product surrounding an intense endurance event reduces pain perception and catabolic stress in the post-exercise period. Further research is necessary to determine long-term supplementation effects with endurance training.

